# Comparing Charlson Comorbidity Index Scores between Anesthesiologists, Patients, and Administrative Data: A Prospective Observational Study

**DOI:** 10.3390/jcm13051469

**Published:** 2024-03-03

**Authors:** Eike J. Röhrig, Henning Schenkat, Nadine Hochhausen, Anna B. Röhl, Matthias Derwall, Rolf Rossaint, Felix Kork

**Affiliations:** 1 Department of Anesthesiology, Medical Faculty, RWTH Aachen University, 52074 Aachen, Germany; eike.roehrig@rwth-aachen.de (E.J.R.); nhochhausen@ukaachen.de (N.H.); anna.roehl@rheinmaasklinikum.de (A.B.R.); matthias.derwall@joho-dortmund.de (M.D.); rrossaint@ukaachen.de (R.R.); 2 Deanery of Studies, Medical Faculty, RWTH Aachen University, 52074 Aachen, Germany

**Keywords:** comorbidity, mortality, Charlson Comorbidity Index, health status indicators, outcome, perioperative care

## Abstract

(1) **Background:** Patients’ comorbidities play an immanent role in perioperative risk assessment. It is unknown how Charlson Comorbidity Indices (CCIs) from different sources compare. (2) **Methods:** In this prospective observational study, we compared the CCIs of patients derived from patients’ self-reports and from physicians’ assessments with hospital administrative data. (3) **Results:** The data of 1007 patients was analyzed. Agreement between the CCI from patients’ self-report compared to administrative data was fair (kappa 0.24 [95%CI 0.2–0.28]). Agreement between physicians’ assessment and the administrative data was also fair (kappa 0.28 [95%CI 0.25–0.31]). Physicians’ assessment and patients’ self-report had the best agreement (kappa 0.33 [95%CI 0.30–0.37]). The CCI calculated from the administrative data showed the best predictability for in-hospital mortality (AUROC 0.86 [95%CI 0.68–0.91]), followed by equally good prediction from physicians’ assessment (AUROC 0.80 [95%CI 0.65–0.94]) and patients’ self-report (AUROC 0.80 [95%CI 0.75–0.97]). (4) **Conclusions:** CCIs derived from patients’ self-report, physicians’ assessments, and administrative data perform equally well in predicting postoperative in-hospital mortality.

## 1. Introduction

A recent estimate suggests that at least 4.2 million people worldwide die within 30 days of surgery [1], making it the third-greatest cause of death with 7.7% of all deaths globally [2].

Besides the immanent risk of the surgical procedure, patients’ comorbidities are one of the most relevant risk factors for perioperative mortality. Different scores exist to evaluate the risk of perioperative morbidity and mortality, like the American Society of Anesthesiologists (ASA) physical status [3] or the Elixhauser Comorbidity Score [4,5,6]. A good compromise due to the poor interrater reliability or complexity of the scores could be the Charlson Comorbidity Index (CCI). The CCI was originally designed by Charlson et al. in 1987 to predict one-year mortality in any patient [7]. While initially designed for the general hospital population, the CCI has been frequently used in surgical contexts [8,9,10,11,12,13,14]. Although sometimes the score has been calculated through a self-report questionnaire [15,16], it has more commonly been calculated from patients’ administrative data [4,5] using one of the existing administrative data adaptations [17,18]. When calculated from administrative data, the CCI is often used in retrospective analyses to account for patients’ comorbidities. However, very little scientific data exist on the validity of the CCI in the perioperative setting in general and, moreover, whether the CCI calculated from administrative data is a suitable instrument to describe and control for comorbidities in retrospective analyses.

We therefore conducted a prospective observational study comparing (1) the prospective CCI from patients’ self-report before and (2) anesthesiologists’ assessment after the consultation with the anesthesiologist in our pre-procedural evaluation center to (3) the CCI calculated from our hospital’s administrative data after patients’ discharge. The secondary aim of our study was to compare the predictive capability for perioperative mortality of these different CCIs.

## 2. Materials and Methods

### 2.1. Ethics and Consent

The data safety monitor of the University Hospital RWTH Aachen and the Ethics Committee of the Medical Faculty, RWTH Aachen University, 52074 Aachen, Germany, approved the study (EK 056/17) on 1 March 2017. Patients were included after informed written consent. The study has been reported in line with the STROCSS criteria [19].

### 2.2. Patients and Setting

In our tertiary care hospital, every patient undergoing elective surgery with anesthesia is scheduled for an appointment in our pre-procedural evaluation center. Besides pre-anesthesiologic evaluation screening, the attending anesthesiologist discusses possible anesthesia plans with the patient and documents informed consent to the proposed anesthesia procedures. All patients aged ≥18 years between 7 March and 13 April 2017 were invited to participate in this prospective study. All invitations took place before consulting with the anesthesiologist. Patients were not invited if they did not speak German or were unable to fill out the questionnaire. There was no sample size calculation; we aimed to include 1000 patients. 

### 2.3. Charlson Comorbidity Index from Two Questionnaires

While waiting for the anesthesiologic consultation, participating patients filled out a paper-based questionnaire (Table S1). Patients were asked to estimate their state of health on a scale of 0 to 100 and to acknowledge the existence of comorbidity items in the CCI. The questionnaires were returned before consulting with the anesthesiologist. The consulting physician was asked to fill out a similar paper-based questionnaire (Table S2). Anesthesiologists had to state their work experience and answer the items of the CCI for the respective patient.

### 2.4. Charlson Comorbidity Index and In-Hospital Mortality from Hospital Administrative Data

In Germany, hospitals are legally bound by the Hospital Reimbursement Act (§21 Krankenhausentgeldgesetz, KHEntgG) to collect specific data from all their patients: all diagnoses in international statistical classification of diseases and related health problems (ICD) codes, patients’ procedures in operation and procedure (Operationen- und Prozeduren-Schlüssel, OPS) codes, as well as data on length of stay and type of discharge. These data are forwarded to the Institute for Reimbursement in Hospitals (Institut für das Entgeldsystem im Krankenhaus, InEK), which calculates case-based compensations. 

From these locally archived data, we abstracted the ICD-10 diagnoses, hospital length of stay, whether the patient underwent out-patient or in-patient surgery, and whether the patient died during the hospital stay. CCIs were calculated from the ICD-10 diagnoses from the hospital data management system using the ‘icd’ package for R, which is based on the algorithm introduced by Quan [18]. 

### 2.5. Statistical Analyses

We prespecified the aims of this study prior to obtaining the ethics committee’s approval of our study. We planned to analyze our data using the descriptive measures of Cohen’s Kappa and Bland–Altman plots. As these measures are merely descriptive and do not test a hypothesis, a sample size calculation was not conducted, but we aimed at including 1000 patients. For these reasons, all analyses must be considered exploratory, and hypothesis tests were conducted post-hoc. Cases with missing data in the questionnaires or administrative data were excluded from the analysis. Although the central limit theorem would apply, we used non-parametric testing and reporting because all three methods to calculate the CCI resulted in heavily skewed distributions. Continuous variables were therefore reported as medians and interquartile ranges, and frequencies as absolutes and percentages. Agreement between continuous variables was assessed by calculating both unweighted and weighted Cohen’s kappa with the ‘psych’ package for R. Comparisons between agreements were performed post hoc, and the ‘psych’ package was conducted. In addition, agreement was graphically assessed using Bland–Altman plots from the ‘BlandAltmanLeh’ package for R. The prediction of binary outcomes from continuous variables was compared by plotting Receiver Operating Characteristics (ROC) using the ‘pROC’ package for R, as were the post-hoc pairwise comparisons of ROC curves. All analyses were conducted using R 4.3.1 for MacOs (www.r-project.org, accessed on 7 July 2023), and figures were created using GraphPad Prism 8 for MacOs. The probability of a type I error of <0.05 was considered statistically significant. Correlations were described as follows: ≦0 no correlation, 0.01–0.20 poor correlation, 0.21–0.40 fair correlation, 0.41–0.60 moderate correlation, 0.61–0.80 good correlation, and 0.81–1.0 perfect correlation [20]. 

## 3. Results

### 3.1. Patients

During the study period, a total of 1580 patients aged 18 and older visited our pre-procedural evaluation center; a total of 1114 was approached for participation in our study; 63 declined to participate; and 1051 were successfully recruited for inclusion in our study. After the exclusion of 44 incomplete datasets, the data of 1007 patients were analyzed (Figure S1).

Patients were 52.1% (*n* = 525) female, and they had a median age of 57 [IQR 39 to 58; Table 1]. 

42.3% (*n* = 426) of the study population underwent outpatient surgery, while more than half of the patients (54.1%; *n* = 581) underwent inpatient surgery. In-hospital mortality was 0.9% (*n* = 5) in the inpatient population. According to the CCI derived from the administrative data, “Malignancy without metastases” was the most common comorbidity with 18.1% (*n* = 182) in our study cohort, followed by “Congestive heart failure” and “Peripheral vascular disease” with 6.5% (*n* = 65) each. The least common comorbidities were “Moderate or severe liver disease” with 0.4% (*n* = 4), “Peptic ulcer disease” with 0.2% (*n* = 2), and “Dementia” with 0.1% (*n* = 1). No patient with “HIV or AIDS” was included in our study (Table 1).

### 3.2. Numerical Score of the Charlson Comorbidity Index

**Figure 1:**
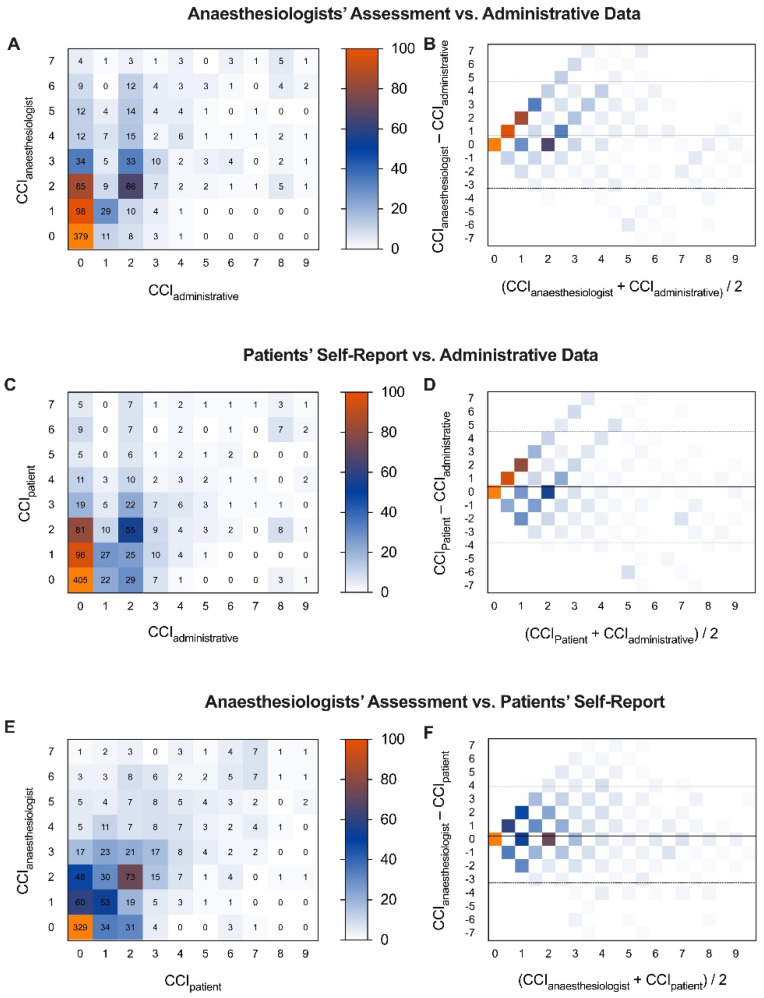
Pairwise comparison of Charlson Comorbidities Indices (CCIs) from anesthesiologists’ assessment (CCIphysician), patients’ self-report (CCIpatient), and administrative data (CCIadministrative) in 1007 surgical patients. On the left, heat maps of correlation, and on the right, heatmap Bland–Altman plots of CCIs derived from (**A**,**B**) anesthesiologists’ assessment and administrative data, (**C**,**D**) patients’ self-report and administrative data, and (**E**,**F**) anesthesiologists’ assessment and patients’ self-report.

First, the numerical scores of the CCI obtained by the anesthesiologists’ assessment (CCIphysician), the patients’ self-report (CCIpatients), and the calculations from the administrative data (CCIadministrative) were compared. The correlation between the anesthesiologists’ assessments and the administrative data was moderate (r = 0.59 (95%CI 0.55 to 0.63); *p* < 0.001; Figure 1A), and the agreement was fair (kappa 0.28, 0.25 to 0.31). The Bland–Altman analyses revealed that anesthesiologists overestimated the CCI score by approximately one point (mean difference 0.74 (limits of agreement −3.19 to 4.68); Figure 1B). Similarly, patients’ self-reports correlated moderately with the administrative data (r = 0.52 (95%CI 0.48 to 0.57); *p* < 0.001; Figure 1C), the agreement was fair (kappa 0.24 (95%CI 0.21 to 0.28)), and patients overestimated their comorbidities by half a point compared to the administrative data (mean difference 0.39 (limits of agreement −3.70 to 4.48); Figure 1D). Anesthesiologists’ assessments and patients’ self-reports correlated the best (r = 0.67 (95%CI 0.63 to 0.70); *p* = 0.001; Figure 1E) and had the best agreement (kappa 0.33 (95%CI 0.30 to 0.37)). Anesthesiologists’ overestimated comorbidities compared to patients’ by less than half a point (mean difference 0.34 (limits of agreement −3.25 to 3.95); Figure 1F and Table S3).

### 3.3. Individual Items of the Charlson Comorbidity Index

The frequency of the individual CCI items from anesthesiologists’ assessments and patients’ self-reports is displayed in Table 2, from the administrative data in Table 1. The agreement of these individual CCI items between anesthesiologists’ assessments, self-reports, and administrative data are shown in Table 2. Overall, most items only show a fair to moderate agreement. 

When comparing the physician’s assessment with the administrative data, agreement was only moderate: “Congestive heart failure” ‘kappa 0.54 (95%CI 0.44 to 0.53)’, “Metastatic solid tumor” ‘ kappa 0.54 (95%CI 0.43 to 0.66)’, and “Malignancy without metastases” ‘kappa 0.51 (95%CI 0.44 to 0.57)’ showed the best agreement. In contrast, “Peptic ulcer disease”, “Dementia”, and “AIDS” showed no agreement (Table 3).

The comparison between the patients’ self-report and the administrative data revealed poorer agreement. Similar to the comparison of the physician’s assessment compared with the administrative data, the best agreement was also found for the two items “Malignancy without metastases” ‘kappa 0.46 (95%CI 0.38 to 0.53)’ and “Metastatic solid tumor” ‘kappa 0.46 (95%CI 0.34 to 0.58)’, followed by “Diabetes without complications” ‘kappa 0.42 (95%CI 0.32 to 0.53)’ and “Renal disease” ‘kappa 0.40 (95%CI 0.30 to 0.51)’ with moderate agreement. No agreement was found for the items “Peptic ulcer disease” and “AIDS” (Table 3).

The best agreements were found when comparing the anesthesiologists’ assessment with the patients’ self-report. Substantial agreement was found for the following items: “Myocardial infarction” ‘kappa 0.70 (95%CI 0.62 to 0.79)’, “Diabetes without complication” ‘kappa 0.63 (95%CI 0.55 to 0.72)’, “Metastatic solid tumor” ‘kappa 0.63 (95%CI 0.55 to 0.72)’, and “Renal disease” ‘kappa 0.63 (95%CI 0.52 to 0.73)’. Again, there was no agreement for the item “AIDS/HIV” (Table 3).

### 3.4. Prediction of In-Hospital Mortality

**Figure 2:**
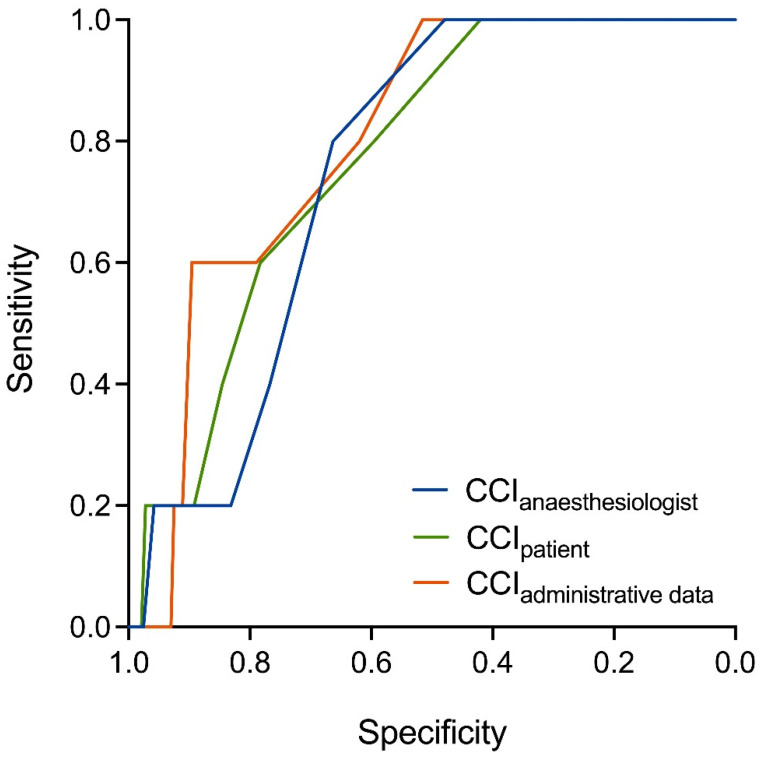
Prediction of surgical in-hospital mortality by three differently derived Charlson Comorbidity Indices (CCIs) in 581 surgical in-patients. The CCI calculated from the administrative data (CCIadministrative) showed the best (AUROC 0.86) predictability for in-hospital mortality. Predictions by the CCIs from the anesthesiologists’ assessment (CCIphysician AUROC 0.80) and the patients’ self-report of comorbidities (CCIpatient AUROC 0.80) are equally good but inferior to the CCI calculated from the administrative data. The pairwise comparison of the ROC curves was not statistically significant (*p* > 0.18).

Figure 2 shows the predictive capabilities of the numerical CCIs for in-hospital mortality in the subgroup of 581 surgical inpatients. The CCI calculated from the administrative data showed the best predictability for in-hospital mortality ‘AUROC 0.80 (95%CI 0.66 to 0.94)’. The predictive capabilities of the CCIs from the anesthesiologists’ assessment and the patients’ self-report were equally good at predicting in-hospital death ‘CCIphysician AUROC 0.75 (95%CI 0.62 to 0.88) and CCIpatient AUROC 0.77 (95%CI 0.61 to 0.93), *p* = 0.88’, but both were inferior compared to the predictive capabilities of the administrative data, although not reaching statistical significance (*p* > 0.58).

## 4. Discussion

In this prospective observational study, we compared three differently collected versions of the CCI in the perioperative setting: one from anesthesiologists’ during pre-procedural assessment, one from patients’ self-report before pre-procedural assessment, and one calculated from the hospitals’ administrative data after patients’ discharge. Regarding the numerical CCI, we found that there was only fair agreement between the anesthesiologists’ assessment and the patients’ self-report when compared with the administrative data. Both the anesthesiologists and the patients overestimated the CCI by 1 and 0.5 points, respectively. The best agreement was found between anesthesiologists’ assessments and patients’ self-reports. When comparing the individual items, “tumor without metastases”, “metastatic solid tumor”, and “congestive heart failure” showed the best agreement in all pair-wise comparisons. Surprisingly, and despite the poor agreement of the three CCIs, all three presented themselves as equally good predictors of in-hospital mortality.

One strength of our prospective observational study is the sample size of 1007, with a fairly even split between inpatients and outpatients (58% vs. 42%), as well as females and males (52% vs. 48%). We explicitly designed the data acquisition in such a way that filling out the questionnaires by patients and anesthesiologists did not coincide. However, we cannot rule out that patients filling out the questionnaire while waiting for consultation with the anesthesiologist influenced patients’ behavior and reporting in their subsequent pre-anesthesiologic consultation.

A high rate of 90% of the approached patients were included in the study, but we could only approach 71% of eligible patients. This has most likely introduced bias in our study. In addition, we only approached patients who were scheduled for elective surgery and who were able to come to our evaluation center either by foot or in a wheelchair. This fact most likely skewed our study population, especially the inpatient subgroup, towards younger and/or physically fitter patients, resulting in lower CCI scores and a lower mortality rate in inpatients. For this reason, fewer “events” may be observed, which may limit the predictability of outcomes. Moreover, the CCI obtained from different sources seems to be a suitable instrument to describe and control for comorbidities in retrospective analyses, whereas no conclusions can be drawn about actual mortality rates. Furthermore, the addition of intraoperative data would result in potential variability in predicting actual mortality. As no intraoperative data were included, this must be seen as a limitation. Additionally, as the questionnaires were in German, non-German speakers were not included. It is possible that a different set of comorbidities may be more prevalent in this group and could lead to different agreements. In addition, the generalizability of these results may be limited because a higher proportion of comorbid patients are treated in German tertiary care university hospitals compared to hospitals of lower care.

Misclassification bias for comorbid conditions may have happened because of inaccurate diagnosis coding. Therefore, administrative data would rather underestimate patients’ comorbidities than misclassify them. This goes in line with the fact that anesthesiologists and patients underestimated the numerical CCI compared to administrative data. Altogether, we therefore assume high data quality.

Our study has two unique aspects compared to other studies. First, we compared three differently collected CCIs, while most other studies compared either patients’ self-report with administrative data [21,22,23,24] or anesthesiologists’ assessment with administrative data [25]. Furthermore, only a few studies compared anesthesiologists’ assessments with patients’ self-reports [25]. We are not aware of any study comparing three different CCIs in a diverse surgical patient population, especially not in a pre-procedural anesthesia evaluation center. The subjects of the most comparable studies were population-based analyses [23,26], emergency patients [21], or patients with arthritis [25]. 

We found that anesthesiologists’ assessments overestimated the CCI from administrative data by approximately one point. This fact may be explained by differing patient perceptions of different CCI sources. While the paper-based questionnaire was filled out by anesthesiologists, the ICD diagnosis codes were added to the administrative data on the surgical wards. It is likely that anesthesiologists focus on cardiopulmonary fitness, whereas surgeons focus on the surgical reason for admission. This conjecture is supported by the fact that the agreement on particular items of the CCI differs. While agreement between the anesthesiologists’ assessment with administrative data on surgical aspects such as malignancies with or without metastases were rather high, agreement was poorer on comorbidities (Table 3) that are well known to be associated with poorer cardiopulmonary fitness and therefore with adverse perioperative outcomes, i.e., diabetes [27,28], renal disease [29,30], and liver disease [9,31].

A similar effect could explain the CCI overestimation of patients’ self-report when compared to administrative data or undercoding in the administrative data compared to patients’ self-report. In fact, other studies have demonstrated similar results. For example, one study found fair to poor agreement when comparing patients’ self-reported CCI with the CCI calculated from administrative data in 520 emergency room patients [21]. The authors explain their findings solely based on the high age of their study population and the unreliable report of comorbidities by the elderly. The median age in our study was indeed lower, but most likely had a similar effect to the one described above: The predominantly surgically driven diagnoses coding for the administrative data could have led to ‘undercoding’ of certain ‘non-surgical’ comorbidities, while, in contrast, patients and anesthesiologists were more meticulous when reporting or detecting these comorbidities. Two facts support this notion. First, single “non-surgical” items with great impact on the numerical CCI score had poor agreement, e.g., diabetes with complications (2 points), or moderate to severe liver disease. Second, anesthesiologists and patients came closest when assessing these comorbidities, especially in contrast to the comparison of either assessment with the administrative data. As a matter of fact, while only 55 patients had diabetes with complications (2 points as an item of the CCI) coded in the administrative data, 88 patients reported this comorbidity, and anesthesiologists detected it in 92 patients (Table 1 and Table 2). The magnitude of this effect is even more pronounced for moderate to severe liver disease (3 points as an item of the CCI): only four patients had diabetes with complications coded in the administrative data, while 28 patients reported it, and anesthesiologists detected it in 33 patients. In addition, these two examples demonstrate why patients’ self-reports and anesthesiologists’ assessments had the best agreement.

The CCI, established in 1987 by Charlson and colleagues as a tool for classifying comorbid conditions [7], has since been validated to assess and adjust for comorbidities in various patient populations [4,15,32]. With the possibility of calculating the CCI automatically from administrative data, it became an easy-to-use, popular tool used in a multitude of studies [5]. However, the CCI has not been validated for all types of patients. To the best of our knowledge, there is no validation for surgical patients. In this manner, we have found that adjusting the weights of the CCI in a sample of 180,000 surgical patients largely improves the predictability of in-hospital mortality in a general surgical population [9]. The following question remained: whether anesthesiologists’ assessments or patients self-reports of CCI items were better compared to administrative data in surgical patients. The results of this study are limited by the small number of events and, in part, the very poor agreement on single items between the different CCI sources. However, the predictability for in-hospital mortality was surprisingly good for all three CCI scores (AUROC ≥0.80). This is well in line with results from a few other studies. A recently published systematic review found 26 studies that predicted mortality from the CCI with an AUROC from 0.66 to 0.90 [33]. Notably, none of the included studies were conducted on surgical patients; most of the studies investigated patients on internal medicine wards and acute geriatric wards. Despite the limitations in our surgical patient sample, the CCI from patients’ self-reports, anesthesiologists’ assessments, or hospital administrative data appears to be equally well suited for the prediction of in-hospital mortality and can therefore be used equally well when adjusting for comorbidities.

In addition to facilitating research in the perioperative patient population, our findings may also have an impact on clinical practice in the future. An automated comorbidity assessment by self-report or from administrative data may improve and expedite the preoperative assessment processes. Anesthesiologists could already be presented with a detailed comorbidity profile of the patient at the first consultation, allowing them to more quickly plan a tailored anesthesiologic and perioperative treatment. Moreover, together with information on the planned surgery and a self-learning model from retrospective hospital data, anesthesiologists and surgeons could be presented with detailed risk estimates for individual patients, allowing them to take individually tailored pre-, intra-, and postoperative interventions or safety measures to improve patients’ outcomes. The inclusion of surgical and anesthetic data could also generate more accurate predictability. Further analyses should illuminate the value of including these data.

## 5. Conclusions

When assessing the predictive capacity of these three CCIs, all performed equally well in predicting in-hospital mortality, with a slight advantage when calculated from administrative data. For that reason, we concluded that CCI from administrative data could be a suitable instrument to control for comorbidities in retrospective data analyses. Of course, the inclusion of intraoperative data would lead to potential variability in the prediction of actual mortality and probably increase accuracy. Agreement of the CCI of anesthesiologists’ assessment with administrative data, patients’ self-reports with administrative data, and patients’ self-report with anesthesiologists’ assessment is only fair. In pair-wise comparisons, patients’ self-report and anesthesiologists’ assessment reached the best agreement. Self-reports, anesthesiologists’ assessments, as well as administrative data, can therefore be used equally well to assess or control for comorbidities in surgical patient populations when researching perioperative outcomes and could also expedite assessment processes and improve patient care in the future when automated and reasonably integrated into routine patient care.

## Figures and Tables

**Table 1:** Characteristics of the study population. Patients (*n* = 1007) were assessed in our pre-anesthetic evaluation center before elective surgery.

Characteristics	Participants (*n* = 1007)	
	*n*, Median	(%), [IQR]
Age, years	57	[39–68]
Sex, female	525	(52.14)
Type of surgery		
Inpatients	581	(57.7)
Outpatients	426	(42.3)
Charlson Comorbidity Index ^a^	1	[0–3]
Malignancy without metastases	182	(18.07)
Congestive heart failure	65	(6.45)
Peripheral vascular disease	65	(6.45)
Chronic pulmonary disease	58	(5.76)
Diabetes without complication	55	(5.46)
Renal disease	51	(5.06)
Metastatic solid tumor	49	(4.87)
Cerebrovascular disease	40	(3.97)
Mild liver disease	26	(2.58)
Myocardial Infarction	19	(1.89)
Hemiplegia	11	(1.09)
Diabetes with chronic complication	10	(0.99)
Rheumatic disease	10	(0.99)
Moderate or severe liver disease	4	(0.40)
Peptic ulcer disease	2	(0.20)
Dementia	1	(0.10)
AIDS	0	(0.00)
Outcome		
In-hospital mortality ^b^	5	(0.5)
Hospital length of stay ^b^, days	5.20	[3.15–10.03]

^a^ numerical Charlson Comorbidity Index and items from administrative data ^b^ of 581 inpatients. AIDS: acquired immunodeficiency syndrome.

**Table 2:** Frequency of Charlson Comorbidity Index items in anesthesiologists’ and patients’ questionnaires in 1007 pre-anesthesiologic consultations.

Charlson Comorbidities	Anesthesiologists		Patients	
Malignancy without metastases	210	(20.85%)	151	(15.00%)
Congestive heart failure	106	(10.53%)	68	(6.75%)
Peripheral vascular disease	113	(11.22%)	116	(11.52%)
Chronic pulmonary disease	136	(13.51%)	95	(9.43%)
Diabetes without complication	92	(9.14%)	88	(8.74%)
Renal disease	107	(10.63%)	89	(8.84%)
Metastatic solid tumor	60	(5.96%)	57	(5.66%)
Cerebrovascular disease	63	(6.26%)	13	(1.29%)
Mild liver disease	28	(2.78%)	31	(3.08%)
Myocardial Infarction	89	(8.84%)	68	(6.75%)
Hemiplegia	13	(1.29%)	10	(0.99%)
Diabetes with chronic complication	24	(2.38%)	16	(1.59%)
Rheumatic disease	47	(4.67%)	59	(5.86%)
Moderate or severe liver disease	33	(3.28%)	28	(2.78%)
Peptic ulcer disease	39	(3.87%)	23	(2.28%)
Dementia	7	(0.70%)	6	(0.60%)
AIDS	1	(0.10%)	2	(0.20%)

AIDS: acquired immunodeficiency syndrome.

**Table 3:** Agreement for physicians’ assessment, patients’ self-report, and administrative data for each item of the Charlson Comorbidity Index (Cohen’s kappa with 95%CI) in 1007 surgical patients.

	Physicians’ Assessment vs. Administrative Data		Patients’ Self-Report vs. Administrative Data		Physicians’ Assessment vs. Patients’ Self-Report	
Malignancy without metastases	0.51	(0.44–0.57)	0.46	(0.38–0.53)	0.50	(0.43–0.57)
Congestive heart failure	0.54	(0.44–0.63)	0.46	(0.21–0.42)	0.50	(0.40–0.59)
Peripheral vascular disease	0.34	(0.24–0.43)	0.46	(0.09–0.26)	0.32	(0.23–0.40)
Chronic pulmonary disease	0.37	(0.28–0.46)	0.39	(0.29–0.49)	0.49	(0.41–0.57)
Diabetes without complication	0.41	(0.30–0.51)	0.42	(0.32–0.53)	0.63	(0.55–0.72)
Renal disease	0.46	(0.36–0.55)	0.40	(0.30–0.51)	0.62	(0.53–0.70)
Metastatic solid tumor	0.54	(0.43–0.66)	0.46	(0.34–0.58)	0.63	(0.52–0.73)
Cerebrovascular disease	0.28	(0.16–0.39)	0.10	(−0.02–0.21)	0.11	(0.01–0.21)
Mild liver disease	0.16	(0.02–0.30)	0.12	(−0.01–0.24)	0.28	(0.13–0.44)
Myocardial Infarction	0.29	(0.18–0.40)	0.32	(0.20–0.45)	0.70	(0.62–0.79)
Hemiplegia	0.24	(0.01–0.47)	0.09	(−0.09–0.26)	0.25	(0.01–0.49)
Diabetes with chronic complication	0.40	(0.19–0.61)	0.22	(0.00–0.44)	0.34	(0.15–0.53)
Rheumatic disease	0.27	(0.12–0.42)	0.16	(0.04–0.28)	0.30	(0.18–0.42)
Moderate or severe liver disease	0.10	(−0.03–0.24)	0.18	(0.00–0.36)	0.44	(0.28–0.60)
Peptic ulcer disease	0.00	(−0.01–0.00)	0.00	(−0.01–0.00)	0.30	(0.15–0.46)
Dementia	0.00	(0.00–0.00)	0.28	(−0.15–0.72)	0.15	(−0.12–0.42)
AIDS	0.00	(0.00–0.00)	0.00	(0.00–0.00)	0.00	(0.00–0.00)

AIDS: acquired immunodeficiency syndrome; CI: confidence interval.

## Data Availability

Data are contained within the article or Supplementary Materials.

## Supplementary Materials

The following supporting information can be downloaded at: https://www.mdpi.com/article/10.3390/jcm13051469/s1. Table S1: Patients questionnaire; Table S2: Anesthesiologists questionnaire; Table S3: Pairwise comparison of the Charlson Comorbidity Index (CCI); Figure S1: Flow chart of patient inclusion.
