# Supplementary material for: Comparing Charlson Comorbidity Index Scores between Anesthesiologists, Patients, and Administrative Data: A Prospective Observational Study
Source: J Clin Med. 2024 Mar 3;13(5):1469. doi: 10.3390/jcm13051469 (PMC10932213; doi:10.3390/jcm13051469)
Supplement: Supplementary file 1 [file jcm-13-01469-s001.zip › Table S1.pdf]

Questionnaire PATIENT

Please complete this questionnaire before consultation with the anesthesiologist.

1) On a scale of 0 to 100, how diseased would you yourself?
(0 = healthy) _________ (100 = sick)

2) Please answer the following questions regarding your health!

a. **How old are you?**
_______ years
b. **What sex are you?**
O female O male
c. **Do you suffer from AIDS?**
O yes O no
d. **Have you ever had a heart attack?**
O yes O no
e. **Do you suffer from congestive heart failure (weakness of the heart muscle)?**
O yes O no
f. **Do you suffer from peripheral vascular disease (e.g. impaired circulation in the legs)?**
O yes O no
g. **Do you suffer from dementia?**
O yes O no
h. **Do you suffer from chronic lung disease?**
O yes O no
i. **Do you suffer from a rheumatic spectrum disease or a connective tissue disease?**
O yes O no
j. **Do you suffer from a gastric or duodenal ulcer?**
O yes O no
k. **Do you suffer from renal disease?**
O yes O no
l. **Do you suffer from leukemia?**
O yes O no
m. **Do you suffer from a malignant lymphoma (cancer of the blood)?**
O yes O no
n. **Do you have any tumor or cancerous disease?**
O with metastases (has the tumor spread?) O no metastases O no
o. **Do you suffer from any disease of your brain blood vessels? Have you ever had a stroke?**
O with hemiplegia O without hemiplegia O no
p. **Do you suffer from liver disease?**
O severe to moderate O mild O no
q. **Do you suffer from Diabetes mellitus?**
O with complications O without complications O no

3) Have you completed this questionnaire on your own?
O yes O No, someone has helped me.

Thank you for your participation!
