# Supplementary material for: Comparing Charlson Comorbidity Index Scores between Anesthesiologists, Patients, and Administrative Data: A Prospective Observational Study
Source: J Clin Med. 2024 Mar 3;13(5):1469. doi: 10.3390/jcm13051469 (PMC10932213; doi:10.3390/jcm13051469)
Supplement: Supplementary file 1 [file jcm-13-01469-s001.zip › Table S2.pdf]

Questionnaire ANESTHESIOLOGIST

Please complete this questionnaire at the end of the pre-anesthesiological consultation.

1) I am

O an anesthesiology specialist, have been for ____ years,
O a resident year ___ of my resedency

2) On a scale of 0 to 100, how diseased would you consider the patient who just consulted you for pre-anesthesiological assessment?

(0 = healthy) _________ (100 = sick)

3) The patient who just consulted me for pre-anesthesiological assessment

a. n/a
b. n/a
c. **suffers from AIDS?**
   O yes O no
d. **has ever had myocardial infarction?**
   O yes O no
e. **suffers from congestive heart failure?**
   O yes O no
f. **suffers from peripheral vascular disease?**
   O yes O no
g. **suffers from dementia?**
   O yes O no
h. **suffers from chronic lung disease?**
   O yes O no
i. **suffers from a connective tissue disease?**
   O yes O no
j. **has a gastric or duodenal ulcer?**
   O yes O no
k. **suffers from renal disease?**
   O yes O no
l. **suffers from leukemia?**
   O yes O no
m. **suffers from a malign lymphoma?**
   O yesO no
n. **has any tumor?**
   O with metastases O without metastasis O no
o. **suffers from cerebrovascular disease?**
   O with hemiplegia O without hemiplegia O no
p. **suffers from liver disease?**
   O severe to moderate O mild O no
q. **suffers from Diabetes mellitus?**
   O with end-organ damage O without end-organ damage O no

Thank you for your participation!
