# Supplementary material for: Comparing Charlson Comorbidity Index Scores between Anesthesiologists, Patients, and Administrative Data: A Prospective Observational Study
Source: J Clin Med. 2024 Mar 3;13(5):1469. doi: 10.3390/jcm13051469 (PMC10932213; doi:10.3390/jcm13051469)
Supplement: Supplementary file 1 [file jcm-13-01469-s001.zip › Table S3.pdf]

Table S3: Pairwise comparisons of the Charlson Comorbidity Index (CCI) by anaesthesiologists' assessment, patients' self-report and from hospital's administrative data: Pearson's correlation coefficient, the intercept and slope from a simple linear regression model, Cohen's kappa for agreement (95%CI) and the mean difference [limits of agreement] from the Bland-Altman plots.

Charlson Comorbidity Index	Anaesthesiologists' assessment vs. administrative data		Patients' self-report vs. administrative data		Anaesthesiologists' assessment vs. Patients' self-report	
Correlation Coefficient	0.65	<0.001	0.55	<0.001	0.71	<0.001
Linear Regression Intercept	1.14	<0.001	0.91	<0.001	0.79	<0.001
Linear Regression Slope	0.65	<0.001	0.55	<0.001	0.71	<0.001
Cohen's Kappa unweighted	0.28	(0.25 – 0.31)	0.24	(0.21 – 0.28)	0.33	(0.30 – 0.37)
Cohen's Kappa weighted	0.56	(-0.31 – 1.00)	0.52	(-0.56 – 1.00)	0.66	(0.35 – 0.97)
Bland-Altman-Mean Difference	0.74	[-3.19 to 4.68]	0.39	[-3.70 to 4.49]	0.35	[-3.25 to 3.95]
