# Supplementary figures and images for: Comparing Charlson Comorbidity Index Scores between Anesthesiologists, Patients, and Administrative Data: A Prospective Observational Study
Source: J Clin Med. 2024 Mar 3;13(5):1469. doi: 10.3390/jcm13051469 (PMC10932213; doi:10.3390/jcm13051469)

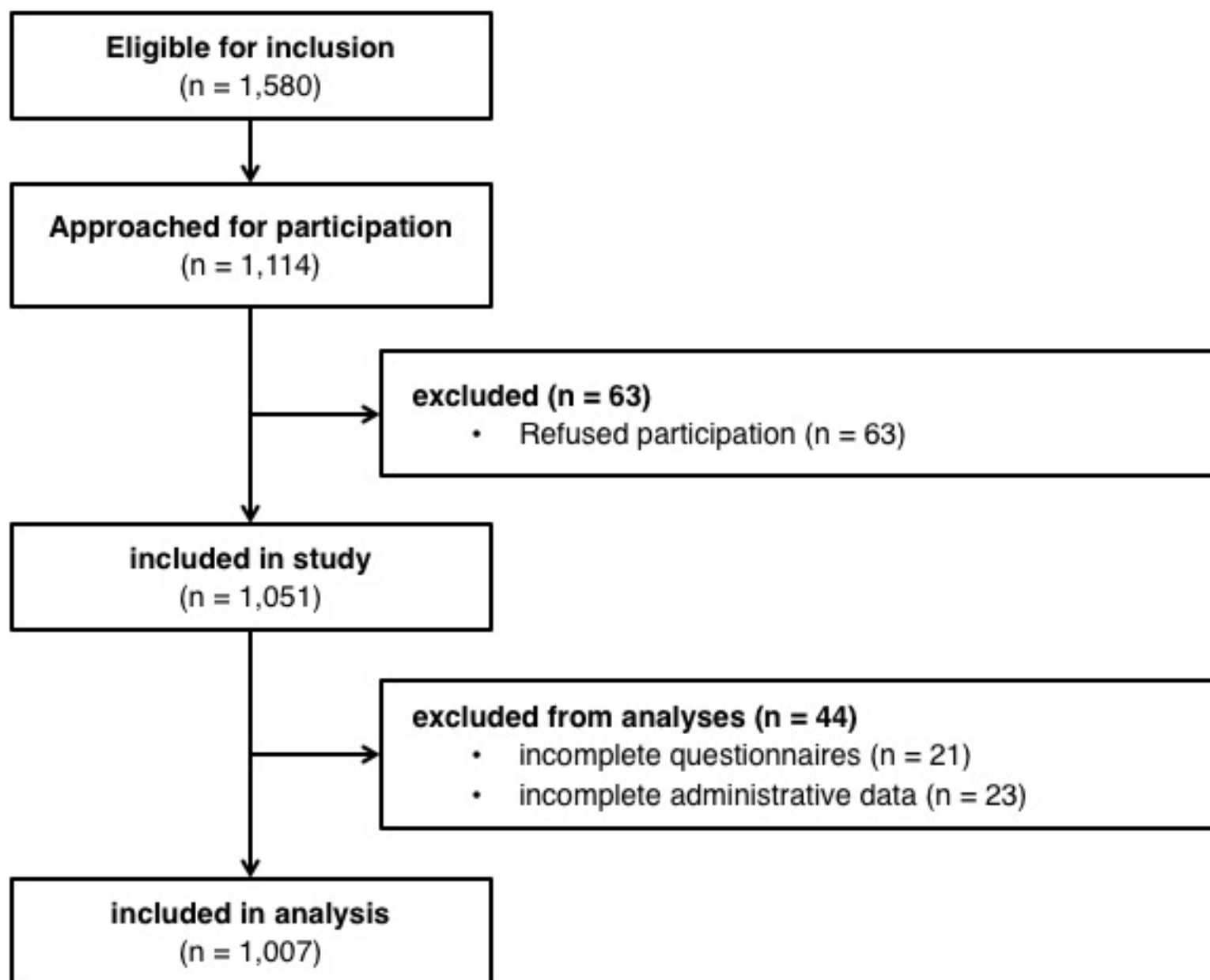

Supplement: Supplementary file 1 [file jcm-13-01469-s001.zip › Figure S1.pdf]
